# Incidence of new-onset HFpEF across CKD stages and Its association with cardio-renal, inflammatory, and fibrotic biomarkers

**DOI:** 10.3389/fcvm.2025.1728415

**Published:** 2026-03-13

**Authors:** Kun Sun, Yao Sun

**Affiliations:** 1 Department of Nephrology, ZiBo Central Hospital, Zibo, Shandong, China; 2 Department of General Practice, ZiBo Central Hospital, Zibo, Shandong, China

**Keywords:** albuminuria, biomarkers, chronic kidney disease, fibrosis, HFPEF, natriuretic peptides, risk prediction

## Abstract

**Background:**

Chronic kidney disease (CKD) substantially increases the risk of heart failure with preserved ejection fraction (HFpEF). In this study, we quantified prospective, stage-specific incidence and evaluated whether biologically anchored, multimarker domains improve prediction beyond clinical factors.

**Methods:**

We conducted a single-center, prospective cohort study (2019–2024) of adults with CKD G1–G5 (not on dialysis) with a 36-month follow-up. Incident HFpEF was adjudicated by two cardiologists using Heart Failure Association—Pre-test assessment, Echocardiography and natriuretic peptide score, Functional testing, Final aetiology (HFA-PEFF) criteria with atrial fibrillation–adjusted natriuretic peptide thresholds, and death was treated as a competing event. Baseline biomarkers were grouped into five domains—cardiac stretch, myocyte injury, fibrosis/remodeling, renal injury/function, and inflammation—and standardized to domain *z*-scores. We estimated incidence per 100 person-years and adjusted associations using Cox and Fine–Gray models. Incremental value was assessed with changes in the C-statistic (*Δ*C), continuous net reclassification improvement (NRI), integrated discrimination improvement (IDI), and calibration, with all metrics bootstrap-validated and false discovery rate control applied for domain tests.

**Results:**

Among 920 participants (mean age 64 ± 12 years; 43% women), 68 HFpEF events occurred over 2,582 person-years, corresponding to 2.63 [95% confidence intervals (CI) 2.01–3.26] events per 100 person-years. Incidence increased with CKD stage—G1 0.29%, G2 1.16%, G3a 1.76%, G3b 3.14%, G4 4.46%, and G5 6.93%—and with albuminuria—A1 1.32%, A2 2.42%, and A3 4.87%. Adjusted hazard ratios were 1.32 (1.17–1.49) per CKD stage and 1.41 (1.20–1.66) per albuminuria category. Per 1 SD, domains associated with higher risk were stretch 1.45 (1.23–1.72), fibrosis/remodeling 1.34 (1.13–1.59), injury 1.26 (1.07–1.48), renal 1.22 (1.04–1.43), and inflammation 1.17 (0.99–1.37). Adding biomarker domains improved discrimination from 0.72 to 0.79 (*Δ*C 0.07, 95% CI 0.03–0.10), with continuous NRI 0.20 and IDI 0.055.

**Conclusions:**

In CKD, HFpEF incidence increases stepwise with CKD stage and albuminuria. Domain-based biomarker profiling, particularly cardiac stretch and fibrosis/remodeling, provides prognostic information beyond clinical factors, supporting a biologically anchored and parsimonious approach to risk stratification.

## Graphical Abstract

**Figure:**
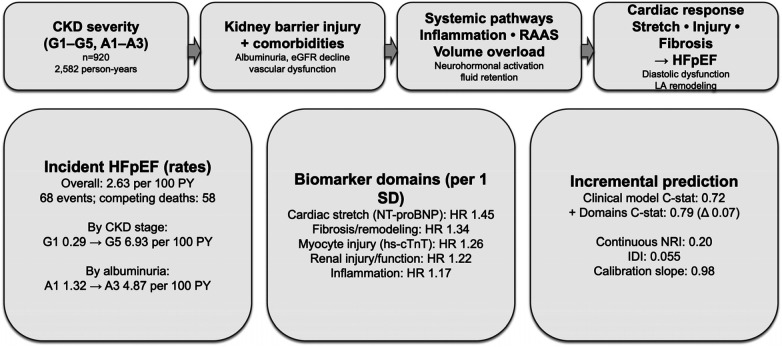
CKD severity, biomarker domains, and incident HFpEF.

## Introduction

Chronic kidney disease (CKD) is a major risk factor for heart failure with preserved ejection fraction (HFpEF). However, prospective data quantifying stage-specific incidence and albuminuria-related gradients remain limited. CKD and HFpEF share common upstream risk factors such as hypertension, diabetes, and obesity (1). The burden of CKD among patients with heart failure is substantial, with studies indicating that approximately 50% of heart failure patients also have CKD (2). The KDIGO guidelines define CKD on the basis of estimated glomerular filtration rate (eGFR) and albuminuria, and these parameters are central to risk assessment and outcome prediction in heart failure (3, 4). Albuminuria, a marker of kidney damage, has been associated with increased risks of mortality and heart failure hospitalization in patients with HFpEF, although its impact appears more pronounced in heart failure with reduced ejection fraction (HFrEF) (3, 5). The ARIC study further demonstrated that both lower eGFR and higher urinary albumin-to-creatinine ratio (UACR) were associated with increased risks of HFpEF and adverse cardiac remodeling (6). However, the specific contributions of CKD stage and albuminuria to incident HFpEF remain incompletely characterized, and some studies suggest that CKD is only modestly associated with overall heart failure without clear differential associations between HFpEF and HFrEF (5). The complex interplay between CKD and HFpEF likely involves systemic inflammation, oxidative stress, and neurohormonal activation (1, 2). Thus, although CKD is widely recognized as a risk amplifier for HFpEF, further research is needed to delineate stage-specific incidence patterns and the prognostic impact of albuminuria (7).

Biomarkers such as natriuretic peptides and cardiac troponins are well-established indicators of myocardial stretch and injury (8, 9). In addition, markers such as soluble suppression of tumorigenesis-2 and growth differentiation factor-15 reflect myocardial remodeling and inflammation, respectively, providing further insight into disease progression (10). Renal dysfunction, a common comorbidity in HFpEF, is often assessed using biomarkers such as albuminuria, which indicates kidney damage and its contribution to overall HFpEF pathophysiology (10). Multi-biomarker panels have been advocated to better capture the biological heterogeneity of HFpEF (11, 12). However, despite their promise, important challenges remain for their routine clinical application (11, 13). Emerging biomarkers derived from omics technologies, including plasma metabolites and circulating microRNAs, are being explored to further enhance diagnostic precision and risk stratification (8, 12). Overall, although substantial progress has been made in identifying biomarkers for HFpEF, further research is required to refine their integration into clinical practice (14).

The current literature reveals substantial knowledge gaps in the prospective characterization of risk across CKD stages G1–G5 and albuminuria categories A1–A3, particularly with respect to the prognostic value of multidomain biomarker panels beyond traditional clinical factors. Although established measures such as estimated glomerular filtration rate (eGFR) and albuminuria are central to risk stratification, they do not fully account for interindividual variability in disease progression (15, 16). Emerging studies suggest that multimarker panels such as proteomic and metabolomic signatures may improve predictive accuracy (17, 18). However, integrating these panels into clinical practice is challenged by issues of assay standardization, reproducibility, and validation across diverse populations (18). Therefore, further research is needed to define the incremental prognostic value of multidomain biomarker panels and to clarify their role in CKD management (19).

While CKD is a known HFpEF risk amplifier, prospective absolute incidence gradients across G1–G5 and A1–A3 and the incremental value of mechanistically grouped biomarker domains beyond clinical factors and baseline HFA-PEFF remain incompletely quantified. We designed this study with three prespecified objectives: first, to quantify the incidence of new-onset HFpEF overall and across CKD stages and albuminuria categories; second, to evaluate the associations of prespecified biomarker domain z scores—capturing cardiac stretch, myocyte injury, fibrosis/remodeling, renal injury/function, and inflammation—with incident HFpEF; third, to test whether adding these domains to a clinical model improves risk prediction, as measured by changes in Harrell's C statistic, continuous net reclassification improvement (NRI), and integrated discrimination improvement (IDI), alongside internal validation and calibration assessment.

## Methods

### Study design and population

We conducted a single-center, prospective observational cohort at ZiBo Central Hospital from April 2019 through October 2024. The protocol was approved by the Ethics Committee of ZiBo Central Hospital before enrollment, and all participants provided written informed consent before the commencement of any study procedures. The study adhered to the ethical principles of the Declaration of Helsinki. Data were managed in accordance with institutional privacy policies and deidentified for analysis, and clinical adjudicators were blinded to biomarker values.

### Inclusion and exclusion criteria

#### Inclusion criteria

Adults aged ≥18 years who received care between April 2019 and October 2024 were eligible if they met a CKD case definition consistent with the KDIGO guidelines and our prespecified operationalization: for G3–G5, an eGFR <60 mL/min/1.73 m^2^ documented on ≥2 occasions at least 90 days apart; for G1–G2, evidence of chronic kidney damage despite eGFR ≥60, defined by albuminuria ≥30 mg/g and confirmed on ≥2 of 3 spot UACR measurements separated by ≥90 days or by other persistent markers of kidney damage (structural abnormalities on imaging or biopsy-proven chronic lesions, or persistent hematuria with dysmorphic red blood cells (RBCs)/RBC casts). Participants were required to be non-dialysis at enrollment, were required to have no prevalent HFpEF at baseline [defined as meeting HFA-PEFF diagnostic criteria (score ≥5, or score 2–4 with confirmatory functional/hemodynamic testing in a symptomatic patient); those with low (≤1) and intermediate (2–4) scores without a final baseline HFpEF diagnosis were eligible], were able to provide written informed consent, and were able to complete baseline clinical assessment and blood/urine sampling for the biomarker panel. When only a single abnormal test was available for G1–G2 and prior documentation was lacking, the participants were flagged as having “unconfirmed chronic damage” and retained in the primary analysis under an intention-to-phenotype approach with a prespecified sensitivity analysis excluding these cases.

#### Exclusion criteria

Individuals were excluded if they did not meet the above CKD definition, were receiving maintenance dialysis at baseline, met HFpEF criteria at baseline adjudication, or were unable to complete baseline biospecimen collection such that the required domain composites could not be computed. Additional exclusions included inability to provide informed consent. These criteria correspond to the flow diagram and account for the observed exclusions because of the factors of not meeting CKD criteria, baseline HFpEF, dialysis at baseline, or missing biomarker data.

### CKD and albuminuria definitions

CKD was defined according to the KDIGO guidelines as abnormalities of kidney structure or function present for ≥3 months, with implications for health, and classified by cause, GFR category, and albuminuria category (C–G–A). Albuminuria categories were A1 (<30 mg/g), A2 (30–300 mg/g), and A3 (>300 mg/g). For G3–G5, CKD required an eGFR <60 mL/min/1.73 m^2^ documented on ≥2 occasions ≥90 days apart. For G1–G2 (eGFR ≥60 mL/min/1.73 m^2^), CKD required confirmed kidney damage, defined by (i) albuminuria ≥30 mg/g on ≥2 of 3 spot UACR measurements separated by ≥90 days, or (ii) other KDIGO-recognized markers of kidney damage (persistent hematuria with dysmorphic RBCs or RBC casts; biopsy-proven glomerular disease with chronic lesions; structural abnormalities on imaging such as polycystic disease, reflux nephropathy, unilateral hypoplasia, or cortical scarring; or persistent tubular disorders). To establish chronicity, we used results obtained at or before enrollment, including EHR laboratory and imaging data within the prior 18 months, provided that two abnormal assessments were ≥90 days apart. When only a single abnormal test was available at enrollment and no prior documentation existed, the participants were flagged as having “unconfirmed chronic damage” and retained in the primary analysis but prespecified for exclusion in sensitivity analyses. Albuminuria and structural criteria for G1–G2, as well as the number and timing of confirmatory measures, were finalized prior to the outcome analysis and are consistent with the KDIGO guidelines.

### Biomarkers and laboratory procedures

A priori, biomarkers were grouped into five biological domains: cardiac stretch (NT-proBNP), myocyte injury (high-sensitivity cardiac troponin T), fibrosis/remodeling (soluble ST2, galectin-3, GDF-15, PIIINP, and TIMP-1), renal injury/function (NGAL, KIM-1, and cystatin C), and inflammation (high-sensitivity C-reactive protein and interleukin-6). Blood and urine samples were processed within 2 h, aliquoted, and stored at −80 °C until batch analysis in a core laboratory using manufacturer-recommended platforms.

All biomarkers were assayed in batch with coefficients of variation <10% and then transformed as needed (natural-log transformation with 1st and 99th percentile winsorization for skewed distributions) and standardized to *z*-scores. For each biological domain, we calculated the unweighted mean of its component biomarker *z*-scores to derive a composite domain score. To place all domains on a common scale for interpretation and model comparability, each composite was then restandardized to a mean of 0 and SD of 1 in the analytic cohort. Accordingly, all reported associations were in accordance with a 1-SD increase in the domain composite.

We prespecified the use of the unweighted mean to preserve biological interpretability and to avoid data-driven overfitting (particularly in the setting of a modest number of events), while acknowledging that domains differ in the number of constituent markers. Therefore, we conducted robustness checks using (1) reliability-weighted composites (weights proportional to 1/assay variance from duplicate runs) and (2) within-domain principal component analysis (PCA), using the first principal component (PC1) scores. Domain internal consistency (Cronbach's *α* and McDonald's *ω* for multimarker domains) and interdomain correlations were estimated to assess coherence and potential collinearity.

### Outcome ascertainment and adjudication

The primary endpoint was incident HFpEF, adjudicated by two board-certified cardiologists blinded to biomarker values using the HFA-PEFF algorithm with atrial fibrillation–adjusted natriuretic peptide thresholds. Intermediate HFA-PEFF scores triggered additional functional testing (stress echocardiography and/or hemodynamic assessment), as available. Discrepancies were resolved by consensus. Adjudication required a left ventricular ejection fraction ≥50% plus structural and/or diastolic functional abnormalities and typical symptoms/signs, with the event date assigned based on clinical documentation (20–22). All-cause death was treated as a competing event in incidence estimation and modeling.

### Follow-up, data sources, and person-time

The participants were contacted at 6–12-month intervals through clinic visits and structured telephone calls. Electronic health records, regional hospital data feeds, and state vital statistics were queried to capture hospitalizations, imaging, and deaths. Loss to follow-up resulted in censoring at the date of last confirmed contact.

### Statistical analysis

Incidence was expressed as events per 100 person-years with exact 95% Poisson confidence intervals (CIs), overall and by CKD stage and albuminuria category. Cumulative incidence over 36 months was illustrated using curves derived from stratum-specific rates under a constant hazard approximation to highlight gradients rather than to provide formal inferences.

Associations with incident HFpEF were estimated using prespecified multivariable Cox proportional hazards models. In the stage model, eGFR was replaced with ordinal CKD stage and the model was adjusted for age, sex, albuminuria category, diabetes, hypertension, atrial fibrillation, and baseline HFA-PEFF score. In the albuminuria model, the UACR was replaced with ordinal albuminuria category and the model was adjusted for age, sex, continuous eGFR, diabetes, hypertension, atrial fibrillation, and HFA-PEFF. In the biomarker domain model, both eGFR and albuminuria category were included along with the same clinical covariates, and hazard ratios were estimated per 1-SD increase in each domain. Proportional hazards assumptions were evaluated using Schoenfeld residuals.

Robustness to the competing risk of death was examined using Fine–Gray subdistribution hazards models, reported alongside cause-specific hazard ratios. Incremental predictive performance of adding the five biomarker domains to the clinical model was assessed using the change in Harrell's C statistic (ΔC) with 95% CIs from 1,000 bootstrap samples, continuous NRI, IDI, and calibration slope with optimism correction via bootstrap refitting. False discovery rate control used the Benjamini–Hochberg procedure applied only to the five domain tests. A two-sided *p* < 0.05 was considered statistically significant. All analyses were conducted in R (version 4.4).

#### Sensitivity analyses and reporting

Sensitivity analyses repeated the association models using Fine–Gray subdistribution hazards, applied stricter HFA-PEFF criteria, recalibrated natriuretic peptide thresholds across eGFR strata, and tested the interaction between the fibrosis/remodeling domain and albuminuria.

## Results

**Figure 1:**
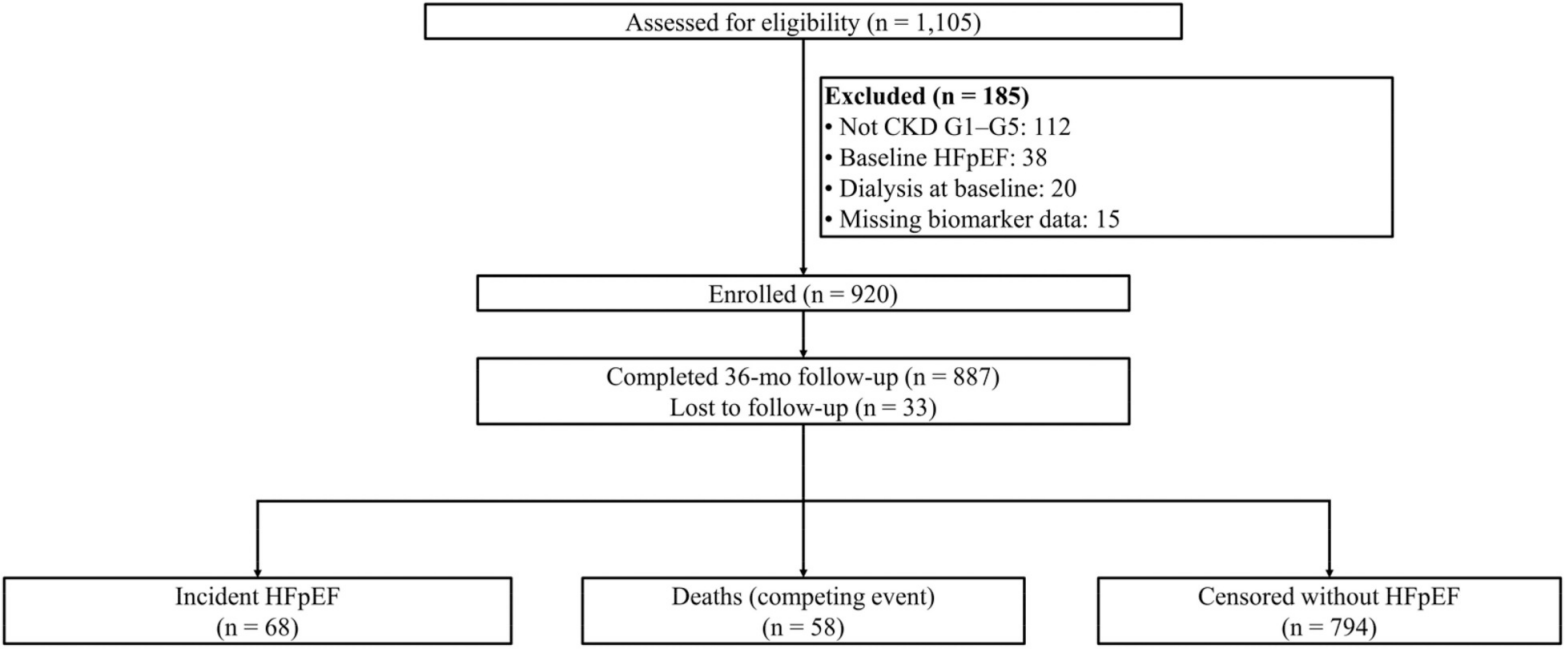
Study flow diagram. Prospective cohort from April 2019 to October 2024. Of 1,105 screened, 920 were enrolled (no baseline HFpEF). Over 36 months, 68 incident HFpEF events occurred; 58 deaths were treated as competing events; 33 were lost to follow-up.

Of 1,105 individuals screened, 920 were enrolled after exclusions for not meeting CKD criteria (*n* = 112), baseline HFpEF (*n* = 38), dialysis at baseline (*n* = 20), and missing biomarker data (*n* = 15), with 887 completing follow-up and the remaining 33 lost to follow-up (Figure 1). The cohort mean age was 64 ± 12 years, 43.0% were women, the median eGFR was 42 [29–63] mL/min/1.73 m^2^, and the median UACR was 110 [24–611] mg/g. The rates of prevalence of diabetes, hypertension, and atrial fibrillation were 46.1%, 88.0%, and 19.0%, respectively. Across CKD stages and albuminuria categories, older age, higher comorbidity burden, and higher biomarker domain z scores accompanied more advanced disease. There were significant linear trends across CKD stages for age, diabetes, hypertension, and atrial fibrillation, and the stretch, injury, fibrosis/remodeling, and renal domains (all *p* < 0.001), with a borderline trend for inflammation (*p* = 0.06), and all five domains increased stepwise across albuminuria categories (all *p* ≤ 0.01) (Table 1).

**Table 1:** Baseline characteristics.

Characteristic	Overall	G1 (*n* = 124)	G2 (*n* = 184)	G3a (*n* = 202)	G3b (*n* = 170)	G4 (*n* = 152)	G5 (*n* = 88)	*p* (trend/overall)
A. By CKD stage (overall N = 920)								
Age, year (mean ± SD)	64 ± 12	60 ± 12	61 ± 12	64 ± 12	66 ± 11	67 ± 11	68 ± 12	<0.001
Women, *n* (%)	396 (43.0)	56 (45.2)	81 (44.0)	88 (43.6)	72 (42.4)	63 (41.4)	36 (40.9)	0.18
eGFR, mL/min/1.73 m^2^ [median (IQR)]	42 [29–63]	95 [90–102]	71 [65–78]	52 [48–56]	36 [32–41]	22 [18–26]	11 [9–13]	<0.001
UACR, mg/g [median (IQR)]	110 [24–611]	25 [7–60]	55 [12–180]	100 [25–450]	210 [60–670]	420 [140–1,110]	650 [260–1,550]	<0.001
Diabetes, *n* (%)	424 (46.1)	45 (36.3)	78 (42.4)	92 (45.5)	82 (48.2)	80 (52.6)	47 (53.4)	<0.001
Hypertension, *n* (%)	810 (88.0)	96 (77.4)	160 (87.0)	181 (89.6)	154 (90.6)	139 (91.4)	80 (90.9)	<0.001
Atrial fibrillation, *n* (%)	175 (19.0)	14 (11.3)	27 (14.7)	37 (18.3)	36 (21.2)	35 (23.0)	26 (29.5)	<0.001
HFA-PEFF score [median (IQR)]	2 [1–3]	1 [1–2]	2 [1–2]	2 [1–3]	2 [2–3]	3 [2–3]	3 [2–4]	<0.001
Domain *z*-scores (mean ± SD)								
Stretch (NT-proBNP)	0.00 ± 1.00	−0.60 ± 0.90	−0.30 ± 0.90	−0.10 ± 0.95	0.20 ± 0.95	0.50 ± 0.95	0.90 ± 0.95	<0.001
Injury (hs-cTnT)	0.00 ± 1.00	−0.50 ± 0.90	−0.20 ± 0.90	0.00 ± 0.95	0.20 ± 0.95	0.40 ± 0.95	0.70 ± 0.95	<0.001
Fibrosis/remodeling	0.00 ± 1.00	−0.40 ± 0.90	−0.10 ± 0.90	0.00 ± 0.95	0.20 ± 0.95	0.50 ± 0.95	0.80 ± 0.95	<0.001
Renal injury/function	0.00 ± 1.00	−0.50 ± 0.90	−0.30 ± 0.90	−0.10 ± 0.95	0.10 ± 0.95	0.60 ± 0.95	1.00 ± 0.95	<0.001
Inflammation	0.00 ± 1.00	−0.10 ± 0.95	−0.05 ± 0.95	0.00 ± 0.95	0.10 ± 0.95	0.20 ± 0.95	0.30 ± 0.95	0.06

Characteristic	A1 (*n* = 377)	A2 (*n* = 294)	A3 (*n* = 249)	*p* (trend/overall)
B. By albuminuria category				
Age, year (mean ± SD)	62 ± 12	64 ± 11	66 ± 11	0.002
Women, *n* (%)	170 (45.1)	125 (42.5)	101 (40.6)	0.09
eGFR, mL/min/1.73 m^2^ [median (IQR)]	60 [45–82]	43 [31–60]	32 [20–49]	<0.001
UACR, mg/g [median (IQR)]	12 [6–20]	110 [60–190]	911 [455–1,560]	<0.001
Diabetes, *n* (%)	139 (36.9)	139 (47.3)	146 (58.6)	<0.001
Hypertension, *n* (%)	315 (83.6)	261 (88.8)	234 (94.0)	<0.001
Atrial fibrillation, *n* (%)	60 (15.9)	55 (18.7)	60 (24.1)	0.002
Domain z-scores (mean ± SD)				
Stretch (NT-proBNP)	−0.30 ± 0.90	0.10 ± 0.95	0.60 ± 0.95	<0.001
Injury (hs-cTnT)	−0.20 ± 0.90	0.10 ± 0.95	0.40 ± 0.95	<0.001
Fibrosis/remodeling	−0.10 ± 0.90	0.20 ± 0.95	0.50 ± 0.95	<0.001
Renal injury/function	−0.40 ± 0.90	0.10 ± 0.95	0.70 ± 0.95	<0.001
Inflammation	−0.05 ± 0.95	0.05 ± 0.95	0.20 ± 0.95	0.01

Among participants with G1 CKD (*n* = 124), 52 (41.9%) met CKD criteria based on confirmed albuminuria (A2/A3 on ≥2 tests), 50 (40.3%) based on imaging- or biopsy-documented structural kidney disease, 9 (7.3%) because of persistent hematuria with dysmorphic RBCs/RBC casts or biopsy-proven glomerulopathy, and 7 (5.6%) because of other KDIGO-recognized markers; 6 (4.8%) participants lacked confirmed chronic damage at enrollment and were flagged as unconfirmed. Among those with G2 CKD (*n* = 184), 124 (67.4%) met CKD criteria by confirmed albuminuria, 33 (17.9%) by structural markers, 9 (4.9%) by hematuria/glomerulopathy, 6 (3.3%) by other markers, and 12 (6.5%) were unconfirmed at enrollment. The median interval between confirmatory assessments for G1–G2 CKD was 147 days (IQR 103–212).

In a sensitivity analysis excluding all unconfirmed G1–G2 participants (*n* = 18; 5.8% of G1–G2), the analytic cohort decreased to *n* = 902 with 2,531 person-years of follow-up. Overall HFpEF incidence changed minimally, from 2.63 to 2.69 events per 100 person-years (95% CI 2.09–3.40). Stage-specific rates for G1 and G2 increased slightly to 0.30 and 1.24 events per 100 person-years, respectively (from 0.29 and 1.16), whereas rates for G3a–G5 remained unchanged.

**Figure 2:**
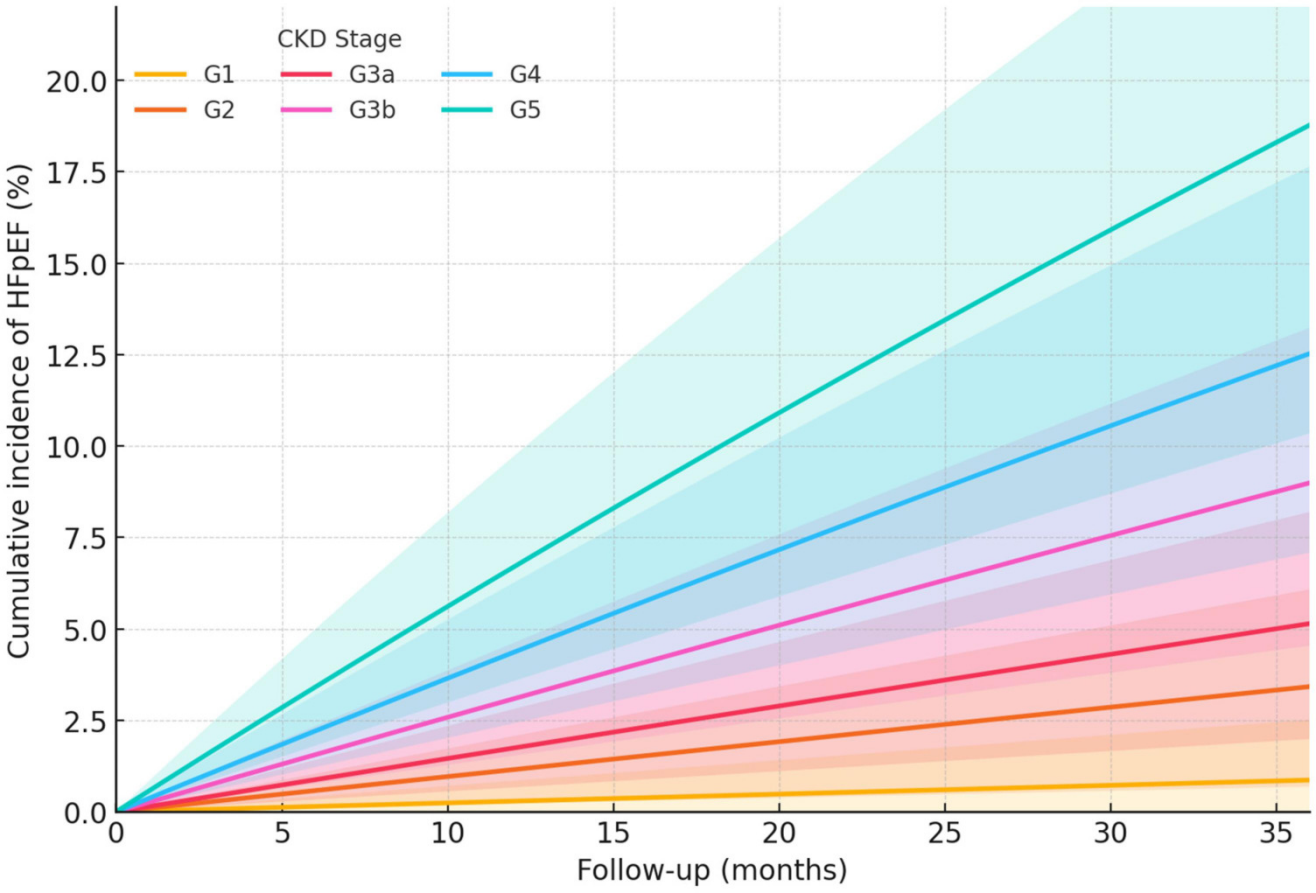
Cumulative incidence of HFpEF by CKD stage. Competing-risk cumulative incidence approximated from stage-specific hazards over 36 months shows a stepwise gradient from G1 to G5. By 36 months, cumulative incidence has increased to ∼0.9% (G1), 3.4% (G2), 5.1% (G3a), 9.0% (G3b), 12.5% (G4), and 18.8% (G5).

**Figure 3:**
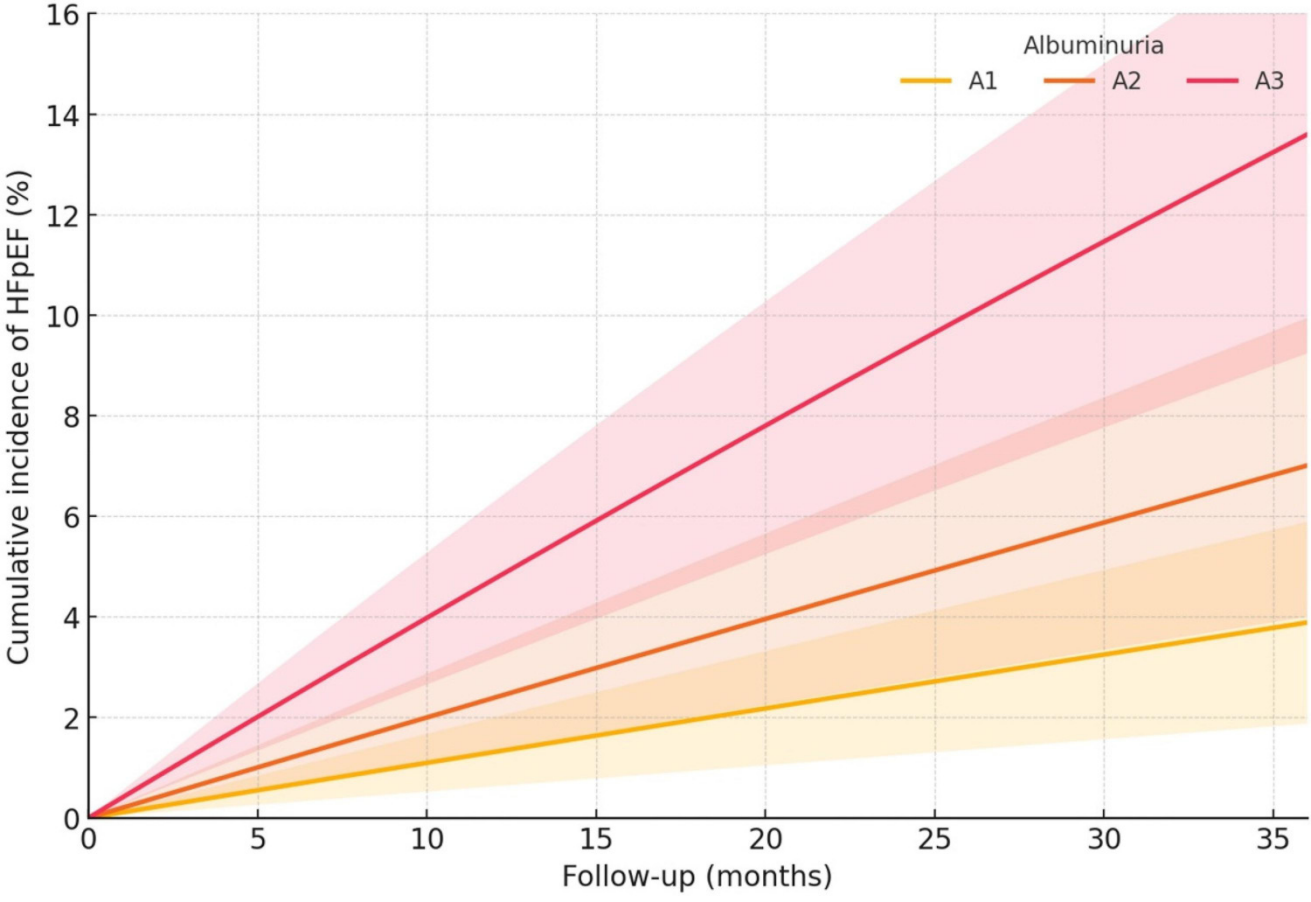
Cumulative incidence of HFpEF by albuminuria category. Higher albuminuria is associated with a greater 36-month risk: ∼3.9% (A1), 7.0% (A2), and 13.6% (A3). Curves derived from category-specific hazards over 36 months.

Over 2,582 person-years, 68 participants developed HFpEF for an overall incidence of 2.63 per 100 person-years (95% CI 2.01–3.26), and 58 deaths occurred as competing events (Table 2). During follow-up, 150 suspected HF presentations were adjudicated; 68 (45.3%) were confirmed as incident HFpEF. Among presentations with intermediate HFA-PEFF scores (2–4), 52/85 (61.2%) underwent additional testing (stress echocardiography 42 and invasive hemodynamics 15). Initial adjudicator disagreement occurred in 14 (9.3%) presentations and was resolved by consensus (Supplementary Table S1). Among confirmed HFpEF events (*n* = 68), exertional dyspnea was documented in 88.2%, peripheral edema in 57.4%, and objective congestion on imaging in 60.3% (Supplementary Table S2). Incidence increased monotonically with CKD severity from 0.29 per 100 person-years in G1 to 6.93 in G5 (G2: 1.16, G3a: 1.76, G3b: 3.14, G4: 4.46), with a highly significant trend (*p*-trend < 0.001). A parallel gradient was evident across albuminuria categories, 1.32, 2.42, and 4.87 per 100 person-years for A1, A2, and A3, respectively, with a *p*-trend < 0.001 (Table 2). The 36-month cumulative incidence curves, generated from the observed stratum-specific rates, visualized a stepwise increase across CKD stages and albuminuria, with approximate risks spanning ≤1% in G1 to about 19% in G5 and rising from ∼4% in A1 to ∼14% in A3 over 36 months (Figures 2, 3).

**Table 2:** Incidence of new-onset HFpEF (competing-risk framework).

CKD stage	*N*	Person-years	HFpEF events	Incidence/100 PY (95% CI)	Deaths (competing)
A. By CKD stage					
G1	124	348.57	1	0.29 (0.00–0.85)	4
G2	184	516.40	6	1.16 (0.23–2.09)	7
G3a	202	568.04	10	1.76 (0.67–2.85)	10
G3b	170	477.67	15	3.14 (1.55–4.73)	13
G4	152	426.03	19	4.46 (2.45–6.47)	14
G5	88	245.29	17	6.93 (3.64–10.23)	10
Overall	920	2,582	68	2.63 (2.01–3.26)	58

Albuminuria	*N*	Person-years	HFpEF events	Incidence/100 PY (95% CI)	Deaths (competing)
B. By albuminuria category					
A1	377	1,058.06	14	1.32 (0.63–2.02)	18
A2	294	825.12	20	2.42 (1.36–3.49)	19
A3	249	698.82	34	4.87 (3.23–6.50)	21
Overall	920	2,582	68	2.63 (2.01–3.26)	58

In multivariable models specified to avoid overadjustment, each one-stage increase in CKD category was associated with a higher risk of HFpEF after adjustment for age, sex, albuminuria category, diabetes, hypertension, atrial fibrillation, and baseline HFA-PEFF score [hazard ratio (HR) 1.32, 95% CI 1.17–1.49; *p* < 0.001]. Similarly, each higher albuminuria category remained independently associated with HFpEF after adjustment for age, sex, eGFR, diabetes, hypertension, atrial fibrillation, and HFA-PEFF (HR 1.41, 95% CI 1.20–1.66; *p* < 0.001). In the biomarker domain model that included both eGFR and albuminuria, higher cardiac stretch (HR 1.45, 95% CI 1.23–1.72; FDR q < 0.001), fibrosis/remodeling (HR 1.34, 95% CI 1.13–1.59; FDR q = 0.002), myocyte injury (HR 1.26, 95% CI 1.07–1.48; FDR q = 0.009), and renal injury/function (HR 1.22, 95% CI 1.04–1.43; FDR q = 0.018) were each significantly associated with incident HFpEF per 1-SD increase, whereas inflammation showed a borderline association (HR 1.17, 95% CI 0.99–1.37; FDR q = 0.058). The direction and magnitude of these associations were consistent in Fine–Gray subdistribution hazard models (Table 3).

**Table 3:** Multivariable associations with incident HFpEF and incremental prediction.

Predictor	Adjusted HR (95% CI)	***p***	FDR ***q*** (domains)	Adjusted sHR (95% CI)	***p*** (sHR)
A. Multivariable associations (cause-specific Cox HRs) and competing-risk robustness (Fine−Gray sHRs)					
CKD stage (per 1-stage increase)	1.32 (1.17–1.49)	<0.001	—	1.28 (1.14–1.45)	<0.001
Albuminuria (A1→A3, per category)	1.41 (1.20–1.66)	<0.001	—	1.37 (1.16–1.62)	<0.001
Biomarker domains (per 1-SD, restandardized composites)					
Stretch (NT-proBNP)	1.45 (1.23–1.72)	<0.001	<0.001	1.41 (1.20–1.66)	<0.001
Injury (hs-cTnT)	1.26 (1.07–1.48)	0.005	0.009	1.24 (1.05–1.46)	0.011
Fibrosis/remodeling	1.34 (1.13–1.59)	<0.001	0.002	1.31 (1.10–1.55)	0.002
Renal injury/function	1.22 (1.04–1.43)	0.014	0.018	1.19 (1.02–1.40)	0.031
Inflammation	1.17 (0.99–1.37)	0.058	0.058	1.14 (0.96–1.35)	0.132

Performance metric	Clinical model	Clinical + domains	Change/value (95% CI)
B. Incremental prediction (clinical model vs. clinical + domains)			
Harrell's C-statistic	0.72 (0.68–0.75)	0.79 (0.76–0.83)	*Δ*C = 0.07 (0.03–0.10)
Continuous NRI	—	—	0.20 (0.08–0.33)
IDI	—	—	0.055 (0.028–0.082)
Calibration slope	—	0.98	0.98 (0.92–1.04)

**Figure 4:**
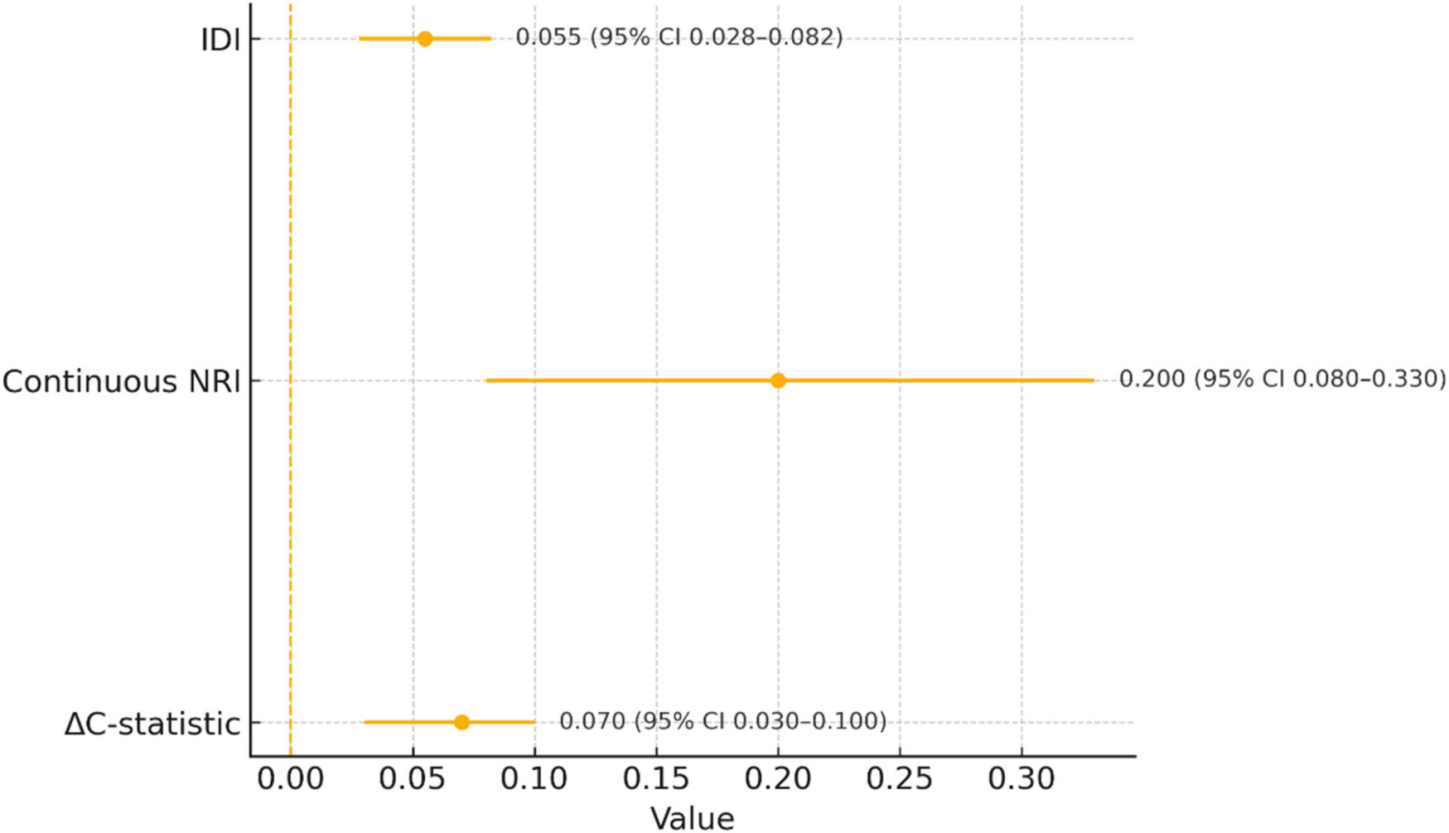
Incremental prediction: clinical vs. clinical + biomarker domains. Point estimates (dots) with 95% CIs (horizontal bars) summarize the improvement when adding domain scores to a clinical model: *Δ*C-statistic 0.07 (0.03–0.10), continuous NRI 0.20 (0.08–0.33), and IDI 0.055 (0.028–0.082). Calibration slope for the augmented model was 0.98 (good agreement).

Adding the five domain scores to the clinical model improved discrimination from 0.72 to 0.79 (*Δ*C 0.07, 95% CI 0.03–0.10), with favorable reclassification (continuous NRI 0.20, 95% CI 0.08–0.33) and risk separation (IDI 0.055, 95% CI 0.028–0.082), while calibration remained excellent (slope 0.98) (Table 3, Figure 4). Sensitivity analyses using Fine–Gray models, stricter HFA-PEFF criteria, and eGFR-stratified natriuretic peptide thresholds yielded concordant results. Interaction testing suggested a stronger association of the fibrosis/remodeling domain in A3 compared with A1/A2 (interaction *p* = 0.041), consistent with the steeper cumulative incidence observed in macroalbuminuria (Table 3, Figures 2, 3).

## Discussion

In this prospective CKD cohort, we observed 68 adjudicated HFpEF events, corresponding to an overall incidence of 2.63 per 100 person-years and a clear stepwise gradient across both CKD stages and albuminuria categories. Domain-based biomarkers provided biologically coherent and statistically robust incremental information. Notably, adding all five domains to the clinical model significantly improved discrimination, reclassification, and overall risk separation while preserving excellent calibration. Taken together, these findings demonstrate a graded burden of new-onset HFpEF across the CKD spectrum and show that the cardiac stretch and fibrosis/remodeling domains confer meaningful incremental prognostic value beyond established clinical factors.

In this CKD cohort, we observed a graded increase in incident HFpEF from G1 to G5 and from A1 to A3, paralleled by the strongest domain-level associations for cardiac stretch and fibrosis/remodeling—a pattern consistent with the microvascular inflammation paradigm of HFpEF, in which hemodynamic load and systemic inflammatory signaling promote myocardial stiffening and extracellular matrix remodeling (23, 24). Mechanistic links in CKD include mineral-bone axis activation that fosters left ventricular hypertrophy and fibrotic remodeling, as well as biomarker signals such as sST2 and galectin-3 that track collagen turnover and adverse remodeling (25–27). Because renal dysfunction elevates natriuretic peptide concentrations through reduced clearance, interpreting “stretch” appropriately in CKD requires renal- and rhythm-adjusted thresholds. Our adjudication strategy using HFA-PEFF with AF-adjusted natriuretic peptide thresholds, together with our domain-based standardization, addresses this challenge and likely contributes to the strong, coherent signals we observed (28, 29). Taken together, these data suggest that integrating hemodynamic load with fibrotic remodeling captures early HFpEF risk in CKD beyond traditional risk markers and single biomarkers (23, 24, 26).

The domain-anchored approach provided incremental prognostic information beyond clinical variables with excellent calibration, supporting a parsimonious strategy to identify CKD patients who merit confirmatory imaging and closer follow-up. In practice, we propose a workflow that begins with standard clinical assessment and HFA-PEFF, uses domain scores to flag high-risk profiles, and prioritizes timely echocardiography when not recently available, repeat/trajectory assessment of diastolic indices, and advanced echocardiographic phenotyping or diastolic stress testing in patients with low/intermediate baseline HFA-PEFF but rising clinical suspicion, particularly in advanced CKD or macroalbuminuria (20, 21, 28, 30, 31). Because albuminuria is itself a powerful vascular risk marker for incident heart failure across eGFR strata, integrating albuminuria with domain scores can rationalize who should undergo natriuretic peptide trending and earlier imaging to unmask evolving HFpEF physiology (32–34).

Because risk was steepest in G4–G5 and A3, a domain-guided strategy complements evidence-based therapies that reduce HF events and slow CKD progression. SGLT2 inhibitors reduce HF events in HFpEF and improve kidney and cardiovascular outcomes in CKD, supporting earlier use in albuminuric and advanced CKD with high domain scores (21, 22, 35–38). Guideline-directed kidney care (KDIGO 2024) remains foundational, including ACEi/ARB therapy for albuminuria. Although spironolactone did not improve the primary outcome in HFpEF and increases the risk of hyperkalemia, finerenone reduces kidney and cardiovascular events in diabetic CKD on background RAS blockade (39–41). From a systems perspective, NT-proBNP–supported triage appears cost-effective in appropriate settings, and SGLT2 inhibitor strategies are increasingly cost-effective for CKD, reinforcing the practicality of a risk-guided pathway that directs imaging, natriuretic peptide monitoring, and therapy to those at greatest risk (42, 43). In sum, parsimonious, domain-anchored risk stratification may help deploy diagnostics and therapies where they are most likely to change outcomes in CKD-associated HFpEF.

Nevertheless, our inferences are qualified by the single-center design and the resulting uncertainty in generalizability, the possibility of residual confounding despite prespecified multivariable adjustment, and biomarker assay variability inherent to multianalyte panels. External transportability is further constrained by restriction to non-dialysis CKD and 3-year follow-up, and the smallest strata yielded wider confidence intervals for stratum-specific incidence estimates. Figures visualizing cumulative incidence relied on a constant-hazard approximation for didactic purposes, with formal inference drawn from cause-specific Cox and Fine–Gray models.

Priorities for future work include multicenter external validation across diverse CKD phenotypes and care settings; pragmatic and health economic evaluation of parsimonious, domain-anchored panels to guide resource allocation; refinement of the fibrosis/remodeling domain in macroalbuminuria, where effect modification was most apparent; and integration of domain scores with advanced imaging and digital phenotyping to enable dynamic, calibrated prediction of HFpEF in CKD and to inform risk-targeted interventions.

In this prospective CKD cohort, the incidence of new-onset HFpEF increased stepwise across CKD stages and albuminuria categories, and a parsimonious, domain-based biomarker strategy—particularly the cardiac stretch and fibrosis/remodeling domains—provided meaningful incremental prognostic value beyond clinical factors, improving discrimination and reclassification while preserving calibration. These findings support targeted risk assessment across the CKD continuum, enabling prioritization of confirmatory imaging, natriuretic peptide monitoring calibrated to renal function and rhythm, and timely initiation of preventive therapies in those at highest risk. External validation and implementation studies are warranted to optimize clinical uptake.

## Data Availability

The raw data supporting the conclusions of this article will be made available by the authors without undue reservation.
