# Supplementary material for: Incidence of new-onset HFpEF across CKD stages and Its association with cardio-renal, inflammatory, and fibrotic biomarkers
Source: Front Cardiovasc Med. 2026 Mar 13;12:1728415. doi: 10.3389/fcvm.2025.1728415 (PMC13021766; doi:10.3389/fcvm.2025.1728415)
Supplement: Supplementary file 1 [file Datasheet1.docx]

**Supplementary Table S1**. **Adjudication workflow, HFA‑PEFF category, additional testing, and consensus**

| **Item** | **n (%)** |
| --- | --- |
| **Analytic cohort** | **920 (100%)** |
| **Confirmed incident HFpEF events (primary endpoint)** | **68 (7.4%)** |
| **Deaths during follow-up (competing events)** | **58 (6.3%)** |
| **Suspected HF/HFpEF presentations screened for adjudication** | 150 (100%) |
| Confirmed incident HFpEF | 68 (45.3%) |
| Not HFpEF (alternative diagnosis) | 74 (49.3%) |
| Indeterminate/insufficient documentation | 8 (5.3%) |
| **Initial adjudicator discordance requiring consensus review** | 14 (9.3%) |
| **HFA‑PEFF category at presentation among suspected cases** |  |
| Low probability (0–1) | 30 (20.0%) |
| Intermediate (2–4) | 85 (56.7%) |
| High probability (≥5) | 35 (23.3%) |
| **Additional functional testing among intermediate HFA‑PEFF (2–4)** |  |
| Intermediate cases with any additional testing performed/reviewed | 52 (61.2%) |
| Stress echocardiography | 42 (49.4%) |
| Invasive hemodynamic assessment | 15 (17.6%) |
| Intermediate cases without additional testing (not available/not performed) | 33 (38.8%) |

**Supplementary Table S2. “Typical symptoms/signs” among confirmed incident HFpEF events**

| **Clinical feature documented at index event** | **n (%) of 68** |
| --- | --- |
| Exertional dyspnea or worsening dyspnea | 60 (88.2%) |
| Orthopnea and/or paroxysmal nocturnal dyspnea | 24 (35.3%) |
| Reduced exercise tolerance/fatigue attributed to congestion | 46 (67.6%) |
| Peripheral edema | 39 (57.4%) |
| Pulmonary rales/crackles | 21 (30.9%) |
| Elevated jugular venous pressure | 14 (20.6%) |
| Objective congestion on imaging (CXR/CT/US report) | 41 (60.3%) |
| Diuretic escalation or IV diuretics at presentation | 50 (73.5%) |
